# Quantification of Bone Fatty Acid Metabolism and Its Regulation by Adipocyte Lipoprotein Lipase

**DOI:** 10.3390/ijms18061264

**Published:** 2017-06-13

**Authors:** Alexander Bartelt, Till Koehne, Klaus Tödter, Rudolph Reimer, Brigitte Müller, Friederike Behler-Janbeck, Joerg Heeren, Ludger Scheja, Andreas Niemeier

**Affiliations:** 1 Department of Biochemistry and Molecular Cell Biology, University Medical Center Hamburg-Eppendorf, Martinistr. 52, 20246 Hamburg, Germany; toedter@uke.uni-hamburg.de (K.T.); br.mueller@uke.uni-hamburg.de (B.M.); f.behler-janbeck@uke.de (F.B.-J.); heeren@uke.uni-hamburg.de (J.H.); l.scheja@uke.uni-hamburg.de (L.S.); 2 Department of Orthopaedics, University Medical Center Hamburg-Eppendorf, Martinistr. 52, 20246 Hamburg, Germany; 3 Department of Osteology and Biomechanics, University Medical Center Hamburg-Eppendorf, Martinistr. 52, 20246 Hamburg, Germany; t.koehne@uke.uni-hamburg.de; 4 Department of Orthodontotics, University Medical Center Hamburg-Eppendorf, Martinistr. 52, 20246 Hamburg, Germany; 5 Electronmicroscopy and Micro-Technology, Heinrich-Pette-Institut for Experimental Virology, Martinistr. 52, 20246 Hamburg, Germany; reimer@hpi.uni-hamburg.de

**Keywords:** bone marrow adipocyte, fatty acids, lipoprotein lipase, osteoblast, bone remodeling

## Abstract

Adipocytes are master regulators of energy homeostasis. Although the contributions of classical brown and white adipose tissue (BAT and WAT, respectively) to glucose and fatty acid metabolism are well characterized, the metabolic role of adipocytes in bone marrow remains largely unclear. Here, we quantify bone fatty acid metabolism and its contribution to systemic nutrient handling in mice. Whereas in parts of the skeleton the specific amount of nutrients taken-up from the circulation was lower than in other metabolically active tissues such as BAT or liver, the overall contribution of the skeleton as a whole organ was remarkable, placing it among the top organs involved in systemic glucose as well as fatty acid clearance. We show that there are considerable site-specific variations in bone marrow fatty acid composition throughout the skeleton and that, especially in the tibia, marrow fatty acid profiles resemble classical BAT and WAT. Using a mouse model lacking lipoprotein lipase (LPL), a master regulator of plasma lipid turnover specifically in adipocytes, we show that impaired fatty acid flux leads to reduced amounts of dietary essential fatty acids while there was a profound increase in de novo produced fatty acids in both bone marrow and cortical bone. Notably, these changes in fatty acid profiles were not associated with any gross skeletal phenotype. These results identify LPL as an important regulator of fatty acid transport to skeletal compartments and demonstrate an intricate functional link between systemic and skeletal fatty acid and glucose metabolism.

## 1. Introduction

The adipose organ is a highly dynamic tissue comprising white, brown and beige adipocytes [1]. While classical white adipocytes are thought to serve mainly as an energy reservoir and can store vast amounts of lipid, brown adipocytes are able to dissipate nutrients in order to produce heat in response to cold stress [2]. In the context of obesity, the capacity of these stores is exhausted, and consequent improper lipid handling leads to inflammation and insulin resistance, promoting the development of metabolic diseases such as type 2 diabetes and atherosclerosis [3]. On the other hand, the activation of brown adipose tissue (BAT) leads to channeling of excess nutrients into brown adipocytes, ameliorating systemic metabolic health [4,5,6]. Next to classical adipose depots such as interscapular, inguinal or gonadal sites, adipocytes are also found in the bone marrow of the skeleton [7]. These bone marrow adipocytes (BMAs) might play important regulatory roles: For example, they appear to serve as negative effectors of hematopoietic activity [8]. In addition, the presence of BMAs seems to have an impact on bone homeostasis in mice [8] and humans [9]. Furthermore, as an endocrine contributor, BMAs are a source of adiponectin [10]. There are also localization-dependent differences in BMAs, as under high-fat diet conditions, the adiposity in the proximal tibiae of mice increases [11], whereas in the marrow cavity of the distal tibia, there appears no such diet-dependent adipose tissue plasticity [12]. However, in vivo evidence regarding contribution of BMAs to bone homeostasis is still scarce. Osteoblasts and adipocytes share a common mesenchymal precursor [13]. The lineage decision is critically dependent on the activity of a transcription factor network, including peroxisome proliferator-activated receptor-γ (PPARγ) [14]. The activation of PPARγ in bone marrow stem cells, for example by application of the synthetic PPARγ agonist rosiglitazone, enhances adipogenesis while impairing osteoblastogenesis in vitro [15] and in vivo [16]. In addition, in osteoclasts, PPARγ is also involved in differentiation [17,18]. It is well accepted that endogenous activation of PPARγ is mediated by certain lipid species, e.g., fatty acids and fatty acid derivatives such as prostaglandins [14]. Like in other cell types, fatty acids in adipocytes are derived both from de novo lipogenesis (DNL) as well as from vascular delivery via lipoproteins [19]. Chylomicrons and very-low density lipoproteins carry triglycerides, cholesterol, lipophilic vitamins as well as other lipids in minor amounts and are the major source of fatty acids stored in adipose tissue. A crucial step in the processing of these triglyceride-rich lipoproteins (TRL) is their binding and processing at the interface between tissue and bloodstream. Here, lipoprotein lipase (LPL), an enzyme predominantly produced by adipocytes, myocytes and macrophages, processes TRL to release fatty acids, which are subsequently internalized by underlying cells or tissues [19]. In addition to free fatty acids, also whole lipoprotein particles are internalized by adipose tissue under certain circumstances [4]. We have shown that TRL are important for vitamin K delivery to osteoblasts and for osteocalcin production [20,21], which is particularly interesting as osteocalcin has been demonstrated to play a role in systemic energy balance [22,23,24]. Furthermore, it has been demonstrated that lipoprotein components and receptors have distinct roles in the regulation of bone mass, for example by manipulating WNT signaling [25], or by yet unidentified mechanisms as in the case of apolipoprotein E [11,26,27]. On the contrary, although osteoblasts are involved in the regulation of energy balance, the contribution of the skeleton to the systemic clearance of nutrients such as fatty acids and glucose remains unclear. In particular, neither whether certain types of fatty acids impact on bone homeostasis nor the identity of their cellular source is well understood. ω-3 fatty acids, especially docosahexaenoic acid (DHA, C22:6n-3), which is abundant in fish oil, have been demonstrated to confer beneficial effects on the skeleton: ω-3 supplementation leads to increased bone mineral density (BMD) in different locations in mice [28], attenuates ovariectomy-induced bone loss [29,30] and in general is positively correlated to bone mineral content in the femur of rats [31]. Largely, these observations can be attributed to specific inhibitory effects of DHA on osteoclasts differentiation as well as activity and on the systemic inflammatory milieu [28,30,32,33]. In addition, conjugated linoleic acids, a family of ω-6 linoleic acid isomers, increase BMD and lower marrow adiposity in ageing, in part by effects on osteoclasts [34,35] but also on osteoblasts [36]. In addition, a class of fatty acid-derivatives, namely *N*-acyl amides have been implicated in bone remodeling through both stimulation of bone formation as well as inhibition of bone resorption [37]. In summary, although several nutritional studies have shown that certain dietary fatty acids are associated with beneficial effects on the skeleton, regulators of postprandial skeletal fatty acid flux have not been identified yet. Possible mechanisms might involve delivery by plasma TRL, which are potentially processed by LPL expressed by BMAs to release essential fatty acids and are subsequently internalized by specialized bone cells. Moreover, when food supply is restricted, BMAs could represent a reservoir of essential lipids as their lipid storage capacity exceeds those of osteoblasts or osteoclasts, in which excessive fatty acid storage might cause lipotoxicity. A balanced local fatty acid milieu might be crucial for healthy bone remodeling whereas an increase in certain deleterious fatty acid species released by BMAs could favor osteoblast dysfunction and bone loss.

Here we quantify the contribution of the skeletal bone compartments to systemic nutrient handling and characterize bone as well as bone marrow fatty acid composition in comparison to established metabolically active tissues. In addition, we address the question whether LPL expressed by adipocytes, including BMAs, is a regulator of bone fatty acid metabolism and skeletal health.

## 2. Results

### 2.1. Lipid Storage in the Skeleton

**Figure 1:**
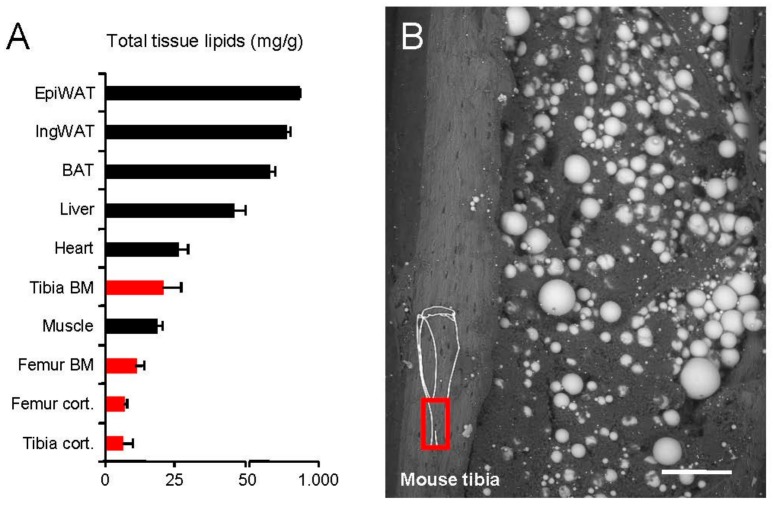
The skeleton is an adipose organ: (**A**) Total lipid content in metabolically active tissues (highlighted in black) and parts of the skeleton (highlighted in red) from 12-week-old female C57BL6/J mice. Mean ± SEM, *n* = 5; (**B**) Environmental scanning electron microscopy of a distal tibial specimen from 12-week-old female C57BL6/J mice revealing both cortical bone as well as adipocyte-containing bone marrow (red box indicates area in the tibia, scale bar: 0.1 mm).

**Figure 2:**
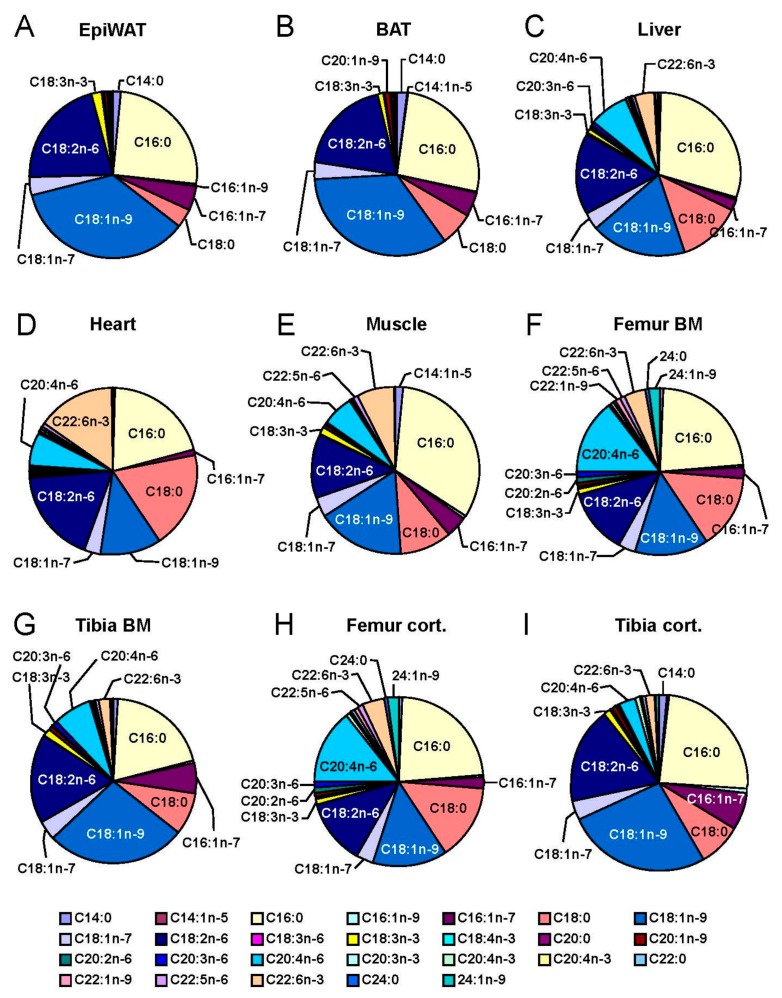
Tibia but not femoral bone marrow resembles adipose fatty acid profiles. Relative fatty acid composition determined by gas chromatography from: (**A**) epididymal white adipose tissue (EpiWAT); (**B**) brown adipose tissue (BAT); (**C**) Liver; (**D**) Heart; (**E**) Muscle; (**F**) femoral bone marrow (Femur BM); (**G**) tibial bone marrow (Tibia BM); (**H**) cortical bone from femur (Femur cort.); and (**I**) cortical bone from tibia (Tibia cort.) from 12-week-old female C57BL6/J mice. Mean, *n* = 7.

In order to compare long bone adiposity to established metabolically active tissues, we isolated specimens from young, lean female C57BL/6J mice and determined total lipid content (Figure 1A). Classical adipose tissue depots, epididymal white adipose tissue (EpiWAT) and subcutaneous inguinal white adipose tissue (IngWAT) as well as interscapular BAT represent tissues that contain predominantly lipids (approximately 400–800 mg/g). In addition, the lipid content of the liver was high (approximately 50 mg/g). Tibial bone marrow (tBM) lipid content was comparable to heart and skeletal muscle tissue. In contrast, in femoral bone marrow (fBM) as well as in tibial and femoral cortical bone, lipids were scarce. To confirm the presence of adipocytes, we performed environmental scanning electron microscopy from the longitudinally cut tibia (Figure 1B). Large, spherical adipocytes were present especially in the distal tibia. We next sought to analyze the fatty acids profiles of long bones in comparison to other metabolically active tissues, in particular adipose tissue. To this end, we isolated total lipids and recorded high-resolution gas chromatography fatty acid profiles (Figure 2). The adipose depots EpiWAT (Figure 2A) and BAT (Figure 2B) contained predominantly oleate (C18:1n-9), linoleate (C18:2n-6) and palmitate (C16:0) but also to minor extent palmitoleate (C16:1n-7), stearate (C18:0), vaccenate (C18:1n-7) and linolenate (C18:3n-3). This composition reflects both dietary essential fatty acids (C18:2n-6 and C18:3n-3) as well as possibly de novo produced fatty acids from e.g., glucose (C18:1n-9, C16:0, C16:1n-7, C18:0 and C18:1n-7). The liver as the major systemic metabolic platform shared this profile largely but was characterized by relatively high levels of arachidonate (C20:4n-6) and C18:0 which are predominantly incorporated in phospholipids and therefore reflects more membrane lipids [38]. Interestingly, the composition of tBM and tibial cortical bone shared some characteristics with adipose tissue, however, with an increased content of membrane-associated fatty acids such as C20:4n-6, 22:6n-3 and C18:0. This membrane-like fatty acid pattern is more pronounced in femur which has the highest C20:4n-6 content of all organs investigated. The ratio of C22:6n-3 to C20:4n-6 in bones is similar to that in liver and unlike that in muscle and heart which have a high C22:6n-3 content. Interestingly, the concentration of nervonic acid (C24:1n-9), a very long-chain fatty acid enriched in sphingolipids, is highest in femur among all tissues. Taken together, while the total amount of lipids per mg tissue is comparatively low in bone, the fatty acid composition, especially in tibia, shares certain characteristics with adipose tissue, possibly reflecting the presence of BMAs. Membrane-enriched fatty acids in bone exhibit a pattern different from all other organs investigated, with a high percentage of C20:4n-6 and C24:1n-9.

### 2.2. The Skeleton Is a Major Target Organ for Dietary Nutrients

**Figure 3:**
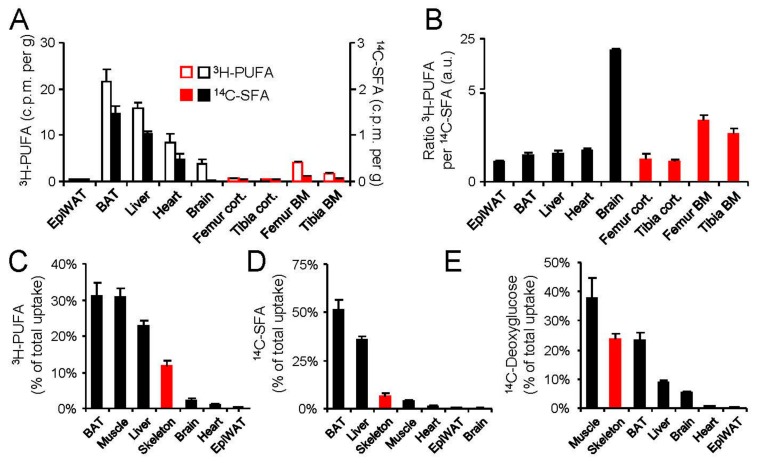
The skeleton significantly contributes to nutrient handling: (**A**) 12-week-old male C57BL6/J mice received an oral fat load mixed with radioactive polyunsaturated acids (^3^H-PUFAs) as well as for saturated fatty acids (^14^C-SFA) tracers; Organ uptake was determined by scintillation counting in isolated tissues. Mean ± SEM, *n* = 7; (**B**) PUFA-to-SFA ratio for metabolically active tissues (highlighted in black) and parts of the skeleton (highlighted in red) calculated from A; (**C**) total organ uptake for metabolically active tissues and the skeleton for SFAs; and (**D**) PUFAs; and (**E**) total organ uptake after an oral glucose tolerance test with tracer amounts of ^14^C-deoxyglucose in 12-week-old male C57BL6/J. Mean ± SEM; *n* = 6 per group. EpiWAT: Epididymal white adipose tissue, BAT: brown adipose tissue, BM: bone marrow.

A major task for metabolically active tissues is on the one hand supplying the body with energy-rich nutrients such as glucose or fatty acids or on the other hand using them for biosynthetic, mechanical or thermogenic means [2]. Moreover, to cope with fasting–feeding cycles, the body has to maintain constant blood glucose when food supply is low or to efficiently store nutrients when they enter the body in the postprandial phase [19]. In order to assess the relative and absolute capacity of the skeleton to participate in the above mentioned nutrient balance, we performed on oral fat load supplemented with tracer amounts of ^3^H-linoleic acid and ^14^C-palmitate (Figure 3A–D). This set-up allows for following the fate of polyunsaturated fatty acids (PUFAs) as well as saturated fatty acids (SFAs) from the meal to target organs as these PUFAs and SFAs can either be transported by lipoproteins or bound to albumin, respectively. The specific SFA-uptake per mg tissue of fBM, tBM as well as cortical compartments was relatively low compared to other metabolically active tissues such as BAT, liver and heart muscle (Figure 3A). In contrast, PUFA-uptake into fBM and tBM was similar to brain, which is a dedicated target organ for essential fatty acid metabolism [39] (Figure 3A). This notion was confirmed when calculating the ratio of PUFA per SFA, placing the brain, fBM and tBM on top of all tissues investigated (Figure 3B). As the skeleton, including the marrow compartments, represents approximately 10% of adult body weight [4] we calculated the specific uptake on a total organ basis. From this perspective, the skeleton is the top-fourth organ responsible for whole body PUFA clearance (Figure 3C). Moreover, considering the distribution of SFAs, we found that the skeleton was among the top three organs next to BAT and liver (Figure 3D). As glucose and fatty acid metabolism in the postprandial phase are closely linked, we sought to address the role of the skeleton in glucose handling and therefore additionally performed an oral glucose tolerance test supplemented with tracer amounts of ^14^C-deoxyglucose (Figure 3E). Here, the skeleton displayed the second highest contribution to systemic glucose clearance. Taken together, the skeleton substantially contributes to systemic nutrient clearance.

### 2.3. Impact of LPL Deficiency on Marrow Lipid Metabolism

**Figure 4:**
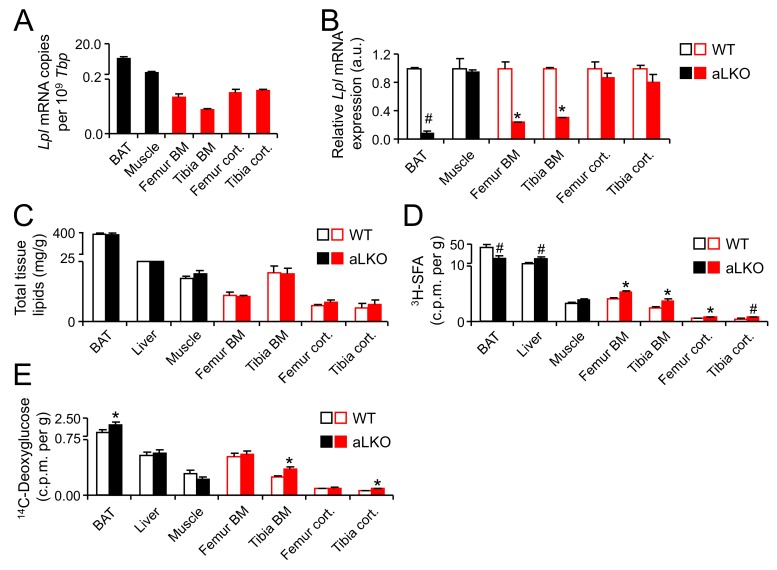
Nutrient handling in aLKO mice: (**A**) Quantitative real-time PCR analysis of *Lpl* expression in metabolically active tissues (highlighted in black) and parts of the skeleton (highlighted in red) in 12-week-old female C57BL6/J mice; and (**B**) adipocyte-specific *Lpl* knock-out (aLKO) mice compared to wild-type controls; (**C**) Total tissue lipids in aLKO mice and controls. Mean ± SEM; *n* = 5 per group; (**D**) Organ uptake of: ^3^H-SFA tracers; and (**E**) ^14^C-deoxyglucose after a combined oral tolerance test. Mean ± SEM; *n* = 6 per group. (* *p* < 0.05, # *p* < 0.01). BAT: brown adipose tissue, BM: bone marrow.

**Figure 5:**
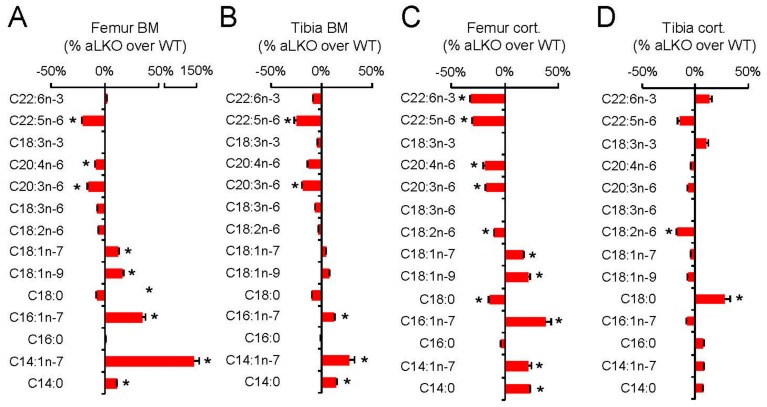
Role of LPL for bone fatty acid composition. Change in relative fatty acid composition determined by gas chromatography from: (**A**) Femur BM; (**B**) Tibia BM; (**C**) Femur cort.; and (**D**) Tibia cort. isolated from 12-week-old female aLKO mice and wild-type controls. Mean ± SEM; *n* = 5 per group. LPL: lipoprotein lipase; BM: bone marrow; aLKO: adipocyte-specific lipoprotein lipase knock-out (* *p* < 0.05).

LPL is the master gatekeeper for the entry of fatty acids from the bloodstream into tissues and is predominantly produced by adipocytes, skeletal and heart muscle cells [19]. Therefore, we hypothesized that, if LPL was also expressed in BMAs and if we genetically deleted LPL in BMAs, LPL deficiency would result in impaired fatty acid delivery to bone marrow and possibly in changes in the fatty acid composition. We have previously shown that the lack of LPL in white and brown adipocytes leads to decreased content of PUFAs in WAT and BAT while there was a profound increase in glucose utilization and DNL-derived fatty acids in adipocytes [40]. In this context we analyzed *Lpl* expression in metabolically active tissue and parts of the skeleton. BAT and muscle tissue displayed higher *Lpl* expression than all parts of the skeleton investigated here (Figure 4A). Surprisingly, we found that in the cortical compartment, *Lpl* was expressed similarly compared to the bone marrow compartment (Figure 4A). Next, we analyzed *Lpl* expression in adipocyte-specific *Lpl* knock-out (aLKO) animals and wild-type controls (Figure 4B), in order to verify the contribution of adipocyte LPL to the respective pools in cortical bone or bone marrow. Whereas in BAT there was a marked decrease in *Lpl* transcript levels, in muscle no effect was detected (Figure 4B), confirming that LPL was specifically deleted adipocytes. In line with an adipocyte-specific *Lpl* deficiency we observe a robust reduction in *Lpl* expression only in marrow but not in cortical bone compartments (Figure 4B). The residual *Lpl* expression in cortical bone is likely due to expression in osteoclasts and other macrophage-like cells [41]. In line with our previous result [40,42], the lack of adipocyte LPL is not associated with alterations in total lipid content of various tissues (Figure 4C). Similarly, in a mixed oral fat and glucose tolerance test using ^14^C-deoxyglucose and ^3^H-C16:0 as tracers, we observed increased flux of deoxyglucose but decreased C16:0 flux into BAT, which is also line with previous results [40,42] (Figure 4D,E). Surprisingly, in the bone compartments, ^3^H-C16:0 uptake was significantly increased in aLKO mice compared to wild-type controls (Figure 4D) whereas ^14^C-deoxyglucose uptake was increased in tibial but not in femoral bone compartments (Figure 4E). In order to investigate the effect on bone fatty acid metabolism of this apparently LPL-independent uptake of SFA, we analyzed the fatty acid composition of femoral and tibial bone marrow versus cortical bone in aLKO and control mice. We found that, in all bone specimens, there was a significant decrease in the abundant dietary essential fatty acid C18:2n-6 or its major conversion product C20:3n-6 (Figure 5), with a similar trend apparent for all ω-6 and most ω-3 fatty acids. This general decrease in PUFAs was associated with a specific increase in fatty acids derived from DNL, especially monounsaturated fatty acids (Figure 5). These effects of LPL deficiency on fatty acid patterns were present in both bone marrow and cortical compartments. The upregulation of de novo synthesized fatty acids was, however, more pronounced in femur (Figure 5). This was most consistent and pronounced for C14:0, which is relatively low in abundance and hence very sensitive to changes in DNL rates. Taken together, lack of LPL in adipocytes lead to a shift from dietary essential fatty acids, PUFAs, to DNL-derived, monounsaturated fatty acids (MUFAs) in both femoral and tibial bone and indicate that lipoprotein catabolism by LPL is an important route for skeletal fatty acid supply to BMAs but possibly also to specialized bone cells.

## 3. Discussion

Here, we show that the skeleton is a major contributor to the clearance of plasma fatty acids and, in line with earlier findings [43], even more so for glucose. Fatty acid compositions of the long bones of the lower extremities differ in a site-specific manner with variations between marrow and cortical compartments as well as between tibia and femur. Tibia bone marrow is characterized by a fatty acid profile that shares characteristics with classical WAT and BAT while the femur (marrow and cortical) is characterized by a particularly high percentage of specific fatty acids (e.g., arachidonic acid and nervonic acid). LPL is required for efficient fatty acid uptake into skeletal compartments as LPL deficiency in adipocytes leads to a shift from dietary essential fatty acids (PUFAs) to DNL-derived, monounsaturated fatty acids (MUFAs) in both femoral and tibial bone. Interestingly, this effect extended from the marrow to the cortical compartment, suggesting that BMA may act as a hub for fatty acid delivery to specialized bone cells.

To the best of our knowledge, this is the first in-depth analysis of both fatty acid compositions of different bone compartments in mice as well as the contribution of the skeleton to systemic fatty acid metabolism. As there are gender-specific differences in systemic nutrient handling in mice, it is important to keep in mind that the present results were obtained in female animals. Thus, in future studies, a thorough comparative analysis of females and males should be added. Bone marrow adipose tissue, which was formerly thought to only represent a passive void filling tissue in ageing bone marrow cavities, is now appreciated as a local and systemic regulator of multiple biological processes such hematopoiesis, osteogenesis and energy metabolism [44]. Whether BMAs share characteristic of classical adipocytes, and if so to which relative degree, is still a matter of debate [45]. The present phenotypic analysis of the fatty acid profiles of bone marrow compared to WAT and BAT (Figure 2A,B,F,G) gives further support to the concept that BMAs share specific characteristics with classical adipocytes. The finding that tBM fatty acid profiles resemble those of BAT and WAT more closely than those of fBM, may be explained by the fact that the tibia is characterized by a higher baseline degree of adiposity and a lower degree of hematopoietic bone marrow cells compared to the femur. Of note, region-specific differences in the responsiveness of BMAs to high-fat diet feeding [11] or caloric restriction [46] have also been described, in that they appear more plastic and regulated in the proximal tibiae and more constitutive, less reactive in the distal tibia. Recently there has been considerable effort to understand glucose metabolism of osteoblasts, but comparatively little is known about osteoblast utilization of fatty acids. It has been shown that fatty acid β-oxidation in osteoblasts is under control of Wnt-Lrp5 signaling [47] but it remains unclear to which degree osteoblasts contribute to the systemic clearance of dietary fatty acids. Even though we have previously demonstrated that postprandial triglyceride-rich lipoprotein (remnants) are being taken up by the skeleton [21], we did not follow the fate of the cargo fatty acids. The present demonstration that the skeleton directly contributes substantially to fatty acid and glucose uptake from the circulation raises the important question, which fatty acid-regulated signals osteoblasts may send back to appetite regulating cells in the central nervous system. In addition, our study also raises the possibility that glucose from the circulation is shuttled into DNL, thus contributing to marrow adiposity. However, it has been shown that high-fat diet leads to a reduction of glucose uptake into skeletal compartments, at least in femur and lumbar vertebrae of male mice [43]. Therefore, and in light of DNL in adipose tissue being an indicator of metabolic health [48] future work should aim at dissecting glucose uptake and DNL in bone cells (osteoblasts, osteocytes and osteoclasts) from that in bone marrow adipocytes. Lipases are critical enzymes in fatty acid metabolism. We have previously shown that hepatic lipase is expressed by osteoblasts and regulates bone mass in mice [49]. In that study, we did not find LPL to be expressed by osteoblasts and consequently focused on LPL produced by BMAs. In the current study we find that adipocyte-specific deletion of LPL profoundly changed the fatty acid flux through bone compartments and induced a shift towards de novo lipogenesis of MUFAs. Adipocyte LPL is particularly important for PUFA uptake as PUFA are predominantly delivered by lipoproteins rather than as free fatty acids [40,42] (which can taken up by LPL-independent mechanisms as well). This remains to be formally proven for marrow adipocytes as well. The residual amount of LPL expression that we find in bone marrow of the aLKO mice (Figure 4B) is likely to be due to expression by myeloid-derived macrophages or osteoclasts [41] but the role of LPL in these cells for bone metabolism is completely unknown and a potential future study subject. In addition to the metabolic characterization of aLKO mice, we also performed preliminary analyses of the bone phenotype in young and lean aLKO and littermate control mice. Neither did plasma levels of calcium, phosphate, alkaline phosphatase, osteocalcin, osteoprotegerin (OPG), receptor activator of nuclear factor-κ B ligand (RANKL) as well as urinary deoxypyridinoline cross-links (DPD) reveal any abnormalities in aLKO mice (Table S1), nor did we detect any differences in structural cortical or trabecular bone parameters or bone mineral density (Table S2). While these data show that changes in fatty acid profiles do not necessarily translate into phenotypic skeletal abnormalities, they may still suggest that structural bone phenotypes may become apparent upon situations of metabolic stress such as malnutrition or high-fat diet feeding-induced insulin resistance.

In conclusion, this study provides first evidence that the skeleton plays a quantitatively important role in systemic fatty acid metabolism and that marrow adipocyte function is a key element in this relationship that deserves further study in mice and men.

## 4. Methods

### 4.1. Mouse Treatments

All animal experiments were approved by the Animal Welfare Officers of University Medical Center Hamburg-Eppendorf (UKE, Hamburg, Germany) and Behörde für Gesundheit und Verbraucherschutz Hamburg (TVA40/15, April 2015). C57BL/6J mice were purchased from Jackson Labs (Bar Harbor, ME, USA) and bred and housed in the animal facility of the University Medical Center Hamburg-Eppendorf at 22 °C with ad libitum access to water and standard laboratory chow diet (Lasvendi, Soest, Germany). Animals carrying floxed alleles of *lipoprotein lipase* (*Lpl*) as well as mice expressing Cre recombinase under control of the *Fabp4* promoter were purchased from Jackson Labs and only littermates were used. This mouse model was previously described and validated [40,42]. Standardized necropsies were performed after 4 h fasting around noon with 12-week old females. Mice were anesthetized with a lethal dose of Ketamine/Xylazine, blood was withdrawn by cardiac puncture and animals were perfused with PBS containing 10 U/mL heparin (Ratiopharm, Ulm, Germany). Organs were harvested and immediately conserved in TRIzol (Invitrogen, Carlsbad, CA, USA) or snap-frozen in liquid N_2_ and stored at −80 °C. Skeletons were mounted, fixed overnight in 4%-formalin and stored in 80%-ethanol. Oral lipid and glucose tolerance test with radiolabelled tracers (Perkin Elmer (Waltham, MA, USA) or Hartmann Analytic (Braunschweig, Germany), respectively) for linoleic acid ([9,10,12,13-^3^H(*N*)]-linoleic acid, 12 kBq/g body weight) and palmitic acid ([1-^14^C]-palmitic acid, 0.62 kBq/g body weight) or glucose (2-desoxy-d-[^14^C]-glucose, 0.62/g body weight) and palmitic acid ([9,10-^3^H(*N*)]-linoleic acid, 12 kBq/g body weight) were performed as described previously [4,50]. The contributions of different organs to whole body clearance are based on previously published percentages [4]. For the unsaturated per saturated index in Figure 3B, we calculated the ratio of specific uptake (cpm/mg).

### 4.2. Analysis of Lipid and Bone Turnover Markers

Plasma triglycerides and cholesterol were determined using commercial kits (Roche, Basel, Switzerland) that were adapted to microtiter plates. For fast performance liquid chromatography (FPLC) pooled plasma was separated using S6-superose columns (GE Healthcare, Little Chalfont, UK) and lipid levels were analyzed in each fraction as described above. Bone turnover markers were measured according to the manufacturers’ instructions: Alkaline phosphatase was determined using the NPP method (Sigma, St. Louis, MO, USA), OPG and RANKL were determined by ELISA (R&D, Minneapolis, MN, USA), OCN was analyzed by an immunoradiometric assay (Immutopic, San Clemente, CA, USA) and DPD/creatinine were determined by ELISA (Quidel, San Diego, CA, USA). Blood glucose levels were measured using AccuCheck Aviva sticks (Roche) as described previously [38].

### 4.3. Quantitative mRNA Expression Analysis

Tissues were immediately placed into RNAlater (Thermofisher, Waltham, MA, USA), incubated over night and then disrupted in TRIzol^®^ (Invitrogen) using a TissueLyser (Qiagen, Hilden, Germany). Total RNA was isolated using NucleoSpin RNA II kit (Macherey & Nagel, Düren, Germany). Complementary DNA was synthesized using SuperScript^®^ III Reverse Transcriptase (Invitrogen). Quantitative real-time PCR reactions were performed on a 7900HT sequence detection system (Applied Biosystems, Foster City, CA, USA) using TaqMan Assay-on-Demand primer sets supplied by Applied Biosystems.

### 4.4. µ-Computed Tomography (CT) Analysis

High-resolution µCT imaging of the right femur and tibia was performed at a resolution of 10 µm per voxel using a Scanco µCT 40 (Scanco Medical, Bassersdorf, Switzerland) according to the guidelines of American Society for Bone and Mineral Research [51,52]. The specific region for cortical bone analysis included a 1-mm-thick section spanning the femur midshaft diaphysis. The cortical bone was delineated automatically with an image analysis algorithm provided by the manufacturer. Standard Scanco software (µCT Evaluation Program version 6.5, Scanco Medical, Bassersdorf, Switzerland) using the distance transformation model was implemented to evaluate cortical bone thickness (µm).

### 4.5. Fatty Acid Analysis and Total Tissue Lipid

Tissue lipid extracts by the Folch method and total tissue fatty acid profiling by gas chromatography were performed as described in detail recently using 50 µL of solvent per mg of tissue [40]. Concentration of individual fatty acids was calculated as % of total fatty acids. Total tissue lipid was calculated as mg total fatty acids per g wet weight tissue.

### 4.6. Statistics

One-way ANOVA or Student’s *t*-test were used for comparison of groups or genotypes. *p* < 0.05 was considered significant. Fatty acid profiles were corrected for multiple testing and significance adjusted accordingly.

## 5. Conclusions

Skeletal compartments significantly contribute to whole body clearance of glucose and fatty acids and display location-specific differences in fatty acid composition. Adipocyte lipoprotein lipase, which is a gatekeeper for the uptake of lipoprotein-derived fatty acids, profoundly affects the uptake and composition of marrow adipocytes in a location-specific manner in the skeleton.

## Supplementary Materials

Supplementary materials can be found at www.mdpi.com/1422-0067/18/6/1264/s1.

## Abbreviations

aLKO	Adipocyte-specific lipoprotein lipase knock-out
BAT	Brown adipose tissue
BMA	Bone marrow adipocyte
BMD	Bone mineral density
EpiWAT	Epididymal white adipose tissue
DHA	Docosahexanoeic acid
DNL	De novo lipogenesis
DPD	Deoxypyridinoline
fBM	Femoral bone marrow
LPL	Lipoprotein lipase
MUFA	Monounsaturated fatty acid
OPG	Osteoprotegerin
PPARγ	Peroxisome proliferator-activated receptor-γ
PUFA	Polyunsaturated fatty acid
RANKL	Receptor-activated nuclear factor κ-B ligand
SFA	Saturated fatty acid
SkeWAT	Skeletal white adipose tissue
tBM	Tibial bone marrow
TRL	Triglyceride-rich lipoproteins
WAT	White adipose tissue
