# Supplementary material for: Quantification of Bone Fatty Acid Metabolism and Its Regulation by Adipocyte Lipoprotein Lipase
Source: Int J Mol Sci. 2017 Jun 13;18(6):1264. doi: 10.3390/ijms18061264 (PMC5486086; doi:10.3390/ijms18061264)
Supplement: Supplementary file 1 [file ijms-18-01264-s001.pdf]

Supplementary Table S1

Plasma parameter	WT	aLKO
Cholesterol (mg/dl)	160 ± 5	148 ± 5
Triglycerides (mg/dl)	111 ± 5	108 ± 4
Alkaline phosphatase (U/L)	225 ± 12	215 ± 13
Osteocalcin (ng/ml)	87 ± 7	90 ± 10
Osteoprotegerin (ng/ml)	1.47 ± 0.19	1.33 ± 0.03
RANKL (pg/ml)	93 ± 10	96 ± 11
DPD per creatinine	12.3 ± 1.3	11.7 ± 2.5
Calcium (mg/dl)	9.72 ± 0.57	9.66 ± 0.67
Phosphate (μM)	13.98 ± 0.55	14.14 ± 0.78

Supplementary Table S2

μCT Skeletal parameters	Femur		Tibia	
	WT	aLKO	WT	aLKO
Ct.th (μm)	158 ± 3	150 ± 2	200 ± 2	194 ± 5
Ct.Porosity (%)	0.52 ± 0.05	0.51 ± 0.02	0.39 ± 0.04	0.37 ± 0.03
TMD (mg HA/ccm)	1,140 ± 16	1,144 ± 4	1,142 ± 5	1,134 ± 3
BV/TV (%)	6.89 ± 0.38	6.20 ± 0.57	9.89 ± 0.38	10.15 ± 0.54
Tb.N (per mm)	3.63 ± 0.09	3.55 ± 0.11	3.81 ± 0.12	3.92 ± 0.19
Tb.Th (μm)	45.0 ± 1.1	42.8 ± 1.2	46.5 ± 1.0	44.8 ± 1.4
Tb.Sp (μm)	276 ± 7	282 ± 8	265 ± 9	257 ± 14
